# Meta-Analysis of Lung Cancer Risk from Exposure to Diesel Exhaust: Study Limitations

**DOI:** 10.1289/ehp.1408482

**Published:** 2014-09-01

**Authors:** Kenny Crump

**Affiliations:** Private Consultant, Ruston, Louisiana, USA

Vermeulen et al. (2014) published a meta-analysis of the risk of lung cancer from exposure to diesel exhaust using data from three case–control studies—two of workers in the trucking industry (Garshick et al. 2012; Steenland et al. 1998) and one of workers in the mining industry (Silverman et al. 2012). Each of the studies quantified diesel exhaust exposure using cumulative exposure to elemental carbon. However, exposures in the trucking studies were lagged 5 years and those in the mining study were lagged 15 years. Vermeulen et al. (2014) applied these data in a linear regression that regressed the log odds ratio (logOR) versus cumulative lagged elemental carbon. They used the regression parameter from this analysis to predict lifetime excess risks for several lifetime occupational and environmental exposure scenarios, and also to predict the fraction of annual lung cancer deaths attributable to diesel exhaust. These excess risk calculations assumed a lag of 5 years.

In their analysis, Vermeulen et al. (2014)inappropriately mixed data from exposures lagged 5 years and 15 years. The assumption of a 5-year lag used in the excess risk calculations is appropriate only if the exposures in all the underlying studies are also lagged 5 years. To obtain some idea of the quantitative effect of this error, I first reran the analysis of Vermeulen et al. (keeping the mixed lags), except that I did not model the dependence among the ORs from the same study. (I did not have access to data needed to model that dependence.) My analysis yielded a regression parameter [0.88; 95% confidence interval (CI): 0.65, 1.11] similar to that obtained by Vermeulen et al. (0.98; 95% CI: 0.55, 1.41). Next, I conducted the same analysis using this model, except I used all 5-year lags, obtaining (0.38; 95% CI: –0.03, 0.96). This analysis consequently yielded a considerably smaller slope, which was not statistically significantly different from 0. The predictions from my analysis were clearly consistent with the underlying ORs all plotted using a 5-year lag, whereas the predictions from the Vermeulen et al. model clearly were not [e.g., the OR at the highest 5-year lagged exposure was 1.25 (95% CI: 0.55, 2.84)]; the model of Vermeulen et al. (2014) predicted an OR of 5.5, and my model predicted an OR of 2.17). Similar results were obtained using a 0-year lag (5-year and 0-year were the only lag data to which we had access).

There are other limitations of the analysis by Vermeulen et al. (2014): Garshick et al. (2012) employed a second measure of diesel exposure (exposure duration), which Vermeulen et al. did not account for in the analysis; and Vermeulen et al. used very crude exposure summaries (e.g., midpoints of exposure intervals).

Estimates of excess risks should be based on the same lag that is common to the underlying studies. Consequently, it would be inappropriate to base public policy regarding exposures to diesel exhaust upon the meta-analysis of Vermeulen et al. (2014).
